# Blood Perfusion in a Full-Thickness Eyelid Flap, Investigated by Laser Doppler Velocimetry, Laser Speckle Contrast Imaging, and Thermography

**Published:** 2018-02-15

**Authors:** Rafi Sheikh, Khashayar Memarzadeh, Christian Torbrand, Jonas Blohmé, Sandra Lindstedt, Malin Malmsjö

**Affiliations:** Lund University, Skane University Hospital, Department of Clinical Sciences Lund, ^a^Ophthalmology; ^b^Urology; and ^c^Cardiothoracic Surgery, Lund, Sweden

**Keywords:** blood perfusion, eyelid, flap, laser Doppler, laser speckle

## Abstract

**Purpose:** The eyelid is commonly dissected and divided in the process of, for example, blepharotomy, entropion repair, or when preparing a full-thickness eyelid flap to reconstruct a tumor defect. No study has yet been conducted to examine how perfusion in an eyelid is affected by dissection, using modern imaging techniques. **Methods:** The eyelid was divided with a 10-mm vertical incision, 5 mm from the medial canthus, and the incision was extended horizontally by 30 mm to provide a full-thickness eyelid. Blood perfusion was measured along the length of the free dissected eyelid using both laser Doppler velocimetry and laser speckle contrast imaging. Tissue temperature was visualized using a high-resolution infrared camera (thermography). **Results:** Measurements using laser speckle contrast imaging showed that blood flow decreased gradually from the pedicel base to the tip of the free dissected eyelid: 83% at 10 mm, stabilizing at 80% at 20 mm from the pedicel base. These results were supported by laser Doppler velocimetry, showing a reduction in perfusion to 67%, 15 mm from the pedicel base. Thermographic imaging showed a corresponding decrease in temperature from the tip to the pedicel base compared with nondissected eyelids. **Conclusions:** Dissection of an eyelid, to provide a full-thickness eyelid flap, results in only a slight decrease in blood flow. The results support the view that plastic surgery of the eyelids is permissive, and the rich vascularization of the eyelid due to the anastomotic network of vessels in the tarsal plate may increase the likelihood of flap survival and surgical success.

The eyelid is commonly dissected and divided in the process of, for example, (i) a blepharotomy procedure for thyroid-associated ophthalmopathy; (ii) a Quickert procedure for correction of involutional entropion in which the eyelid split transversally, to prevent the upward movement of the preseptal orbicularis, combined with horizontal lid shortening to correct excess lid laxity, and everting sutures to shorten the retractors^1^; (iii) a Tenzel semicircular rotational flap for reconstruction of a full-thickness lid defect by advancing a flap from the lateral canthus^2^; or (iv) a “switch flap” that involves switching a full-thickness eyelid flap on a pedicel (with tarsus, lid margin, and eyelashes), from lower lid to upper lid, or vice versa, in order to reconstruct a tumor defect.^3^ No study has yet been conducted to examine how blood perfusion in an eyelid is affected by such dissection, using modern imaging techniques.

During recent decades, several imaging techniques have been developed providing high-resolution images of the structure and function of tissue. Laser Doppler velocimetry (LDV) is an established technique used for measuring blood flow in plastic surgery. However, to get reproducible results, the technique is best used with invasive probes and it is only possible to study the blood flow in a very small region (1 mm^3^ surrounding the probe, with the technique used in this study).^4^ A more recent technique is laser speckle contrast imaging (LSCI), which produces representative images of the blood flow in the surface of tissue over a larger area (24 × 24 cm^2^, with the technique used in this study). Not only is this technique noninvasive but is also highly reproducible. LSCI is now an established technique in, for example, experimental vascular brain research and plastic surgery but has not been applied in the field of periorbital plastic surgery. Thermography, employing a high-resolution infrared (IR) camera, is another noninvasive technique that measures the temperature of the tissue, which can be used as a proxy for blood flow. This method is suitable for clinical use but has not previously been applied to the study of periorbital skin.

The aim of this study was to investigate perfusion in a full-thickness eyelid flap on a pedicel by using modern imaging techniques. The study was conducted on pigs, which are considered to be a suitable model for dermal and plastic surgery studies, as the epidermis, dermis, and subcutaneous fat resemble those in humans.^5^^,^^6^ The microvascular perfusion was measured using both LDV and LSCI, and the tissue temperature was visualized using thermography.

## METHODS

### Animals and anesthesia

Eight pigs with a body weight of 70 kg were fasted overnight but had free access to water. An intramuscular injection of xylazine (Rompun vet. 20 mg/mL; Bayer AG, Leverkusen, Germany; 2 mg/kg) mixed with ketamine (Ketaminol vet. 100 mg/mL; Farmaceutici Gellini S.p.A., Aprilia, Italy; 20 mg/kg) was used for premedication. Anesthesia was then induced with intravenous sodium thiopental (Pentothal; Abbot Scandinavia, Stockholm, Sweden; 4 mg/kg) and fentanyl (Leptanal; Lilly, France; 2 μg/kg) and maintained by continuous infusion of fentanyl in Ringer's acetate (3.5 µg/kg/h) in combination with sodium thiopental (∼2.5 mg/kg). The animals were orally intubated with cuffed endotracheal tubes. Mechanical ventilation was established in the volume-controlled mode with 35% oxygen (Siemens-Elema AB, Solna, Sweden). The ventilation settings were identical for all animals: respiratory rate 15 breaths/min and minute ventilation 12 L/min. A positive end-expiratory pressure of 5 cm H_2_O was applied. A Foley catheter was inserted into the urinary bladder through a suprapubic cystostomy. Following anesthesia and the surgical procedures, the pig was allowed to stabilize for 1 hour before the experiments were started.

### Ethics

The experimental protocol for this study was approved by the Ethics Committee for Animal Research at Lund University, Lund, Sweden. The research adhered to the tenets of the Declaration of Helsinki as amended in 2008. The animals were also used for other experiments that were considered not to have an impact on the present study.

### Experimental procedure

**Figure 1:**
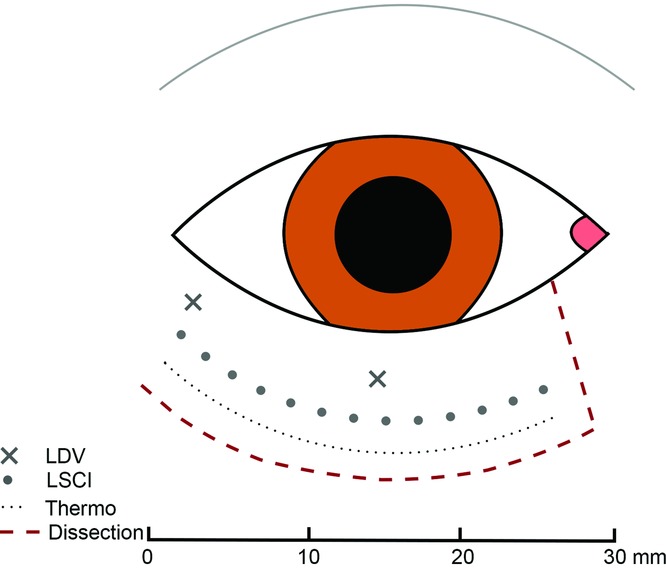
Schematic illustration of the location of the measurement points along the length of a full-thickness eyelid flap (dissection). Blood perfusion was measured using LDV and LSCI. Tissue temperature (Thermo) was measured with a high-resolution infrared camera. LDV indicates laser Doppler velocimetry; LSCI, laser speckle contrast imaging.

The eyelid was divided with a 10-mm vertical incision at 5 mm from the medial canthus, and the incision was extended 30 mm horizontally (Fig 1) to produce a full-thickness eyelid flap on a pedicel. Both upper and lower eyelids were used in the study and the results presented together. It could be argued that the results for the 2 eyelids should be considered separately. However, there were no difference in the results between the upper and lower eyelids and conclusions drawn are considered a true reflection of the actual situation. Perfusion was imaged by LDV, LSCI, and thermography at different measurement points as shown in Figure 1.

### Perfusion measurements

#### Laser Doppler velocimetry

Perfusion was measured by LDV. The technique is based on the emission of a beam of laser light. The light is scattered and partly absorbed by the studied tissue. Light that hit moving blood cells undergoes a change in wavelength (Doppler shift), whereas light hitting static tissue is unchanged. The magnitude and frequency distribution of these changes in wavelength is presumed to be related to the number and velocity of blood cells. A filament probe (MT A500-0, straight microtip with slanted microtip; Perimed AB, Stockholm, Sweden) was inserted at measurement points in the flap base (0 mm) and 15 mm from the flap base (Fig 1), using a 22-G Venflon infusion cannula. The technique enables perfusion in a 1-mm^3^ volume surrounding the tip of the probe.^4^ The filament probe was attached to a master probe (Probe 418), which was then connected to the main LDV unit (Perimed PF5010 unit).

#### Laser speckle contrast imaging

A LSCI system (PeriCam PSI NR System, Perimed AB, Stockholm, Sweden) was used to obtain images of perfusion over the surface of a larger tissue area (24 × 24 cm). The system uses a 785-nm invisible laser beam that is spread over the skin surface by a diffuser creating a speckle pattern. A speckle pattern is the pattern of dark and bright areas formed by random interference in the backscattered light of the area illuminated by laser. Blood perfusion is calculated by analyzing the variations in the speckle pattern. The speckle pattern is recorded in real time at a rate of up to 100 images per second, with a high resolution of up to 100 μm/pixel.

#### Thermography

Thermal energy (electromagnetic radiation) is emitted by all objects at temperatures above absolute zero, and the amount of radiation increases with temperature. Tissue temperature (thermal radiation) measurements were performed using a high-resolution IR camera (FLIR A655sc; FLIR Systems AB, Danderyd, Sweden). The IR camera was a focal plane array, uncooled microbolometer with 640 × 480 pixel resolution, and a thermal sensitivity/NETD (noise equivalent temperature difference) of less than 0.05°C at + 30°C/50 mK. The IR camera was placed approximately 50 cm above the animal, on a Manfrotto 244 Variable Friction Magic Arm mounted on a Manfrotto 190 series tripod. The software ThermaCAM Researcher Pro 2.10 from FLIR Systems was installed in a PC laptop and used to capture the IR images and for data postprocessing. The IR camera was connected to the laptop through the Ethernet interface using a 2-m shielded Ethernet cable. To prevent the conductance of heat from the underlying tissue, an isolating pad was placed under the eyelid.

### Calculations and statistics

Blood perfusion measured with LDV and LSCI is expressed in the arbitrary unit, perfusion units (PUs). Tissue temperature is expressed in degree Celsius (°C). Perfusion and temperature were also calculated as a percentage of the value in the pedicel base (0 mm). The results are presented as median values and ranges. The distance from the pedicel base at which the perfusion and temperature reached a plateau was analyzed using nonlinear regression analysis (1-phase decay) and expressed as median values and 95% confidence intervals. The results are given in scatter plots or box-and-whisker plots in which the box is the 25th to 75th percentiles, the whiskers are the minimum and maximum values (range), and the line indicates the median. Eight pigs were used in this study. For practical reasons, not all experiments were performed in all pigs or all eyelids. The number of experiments (n) is therefore given in the text and figures. Statistical analysis was performed using the Wilcoxon matched-pair test for single comparisons and the Friedman matched-pair test with Dunn's posttest for multiple comparisons. All *P* values in the interval .001 to .300 are written out, whereas other *P* values are given as *P* < .001 and *P* > .30. Calculations and statistics were performed using GraphPad PRISM 7.0a software.

## RESULTS

**Figure 2:**
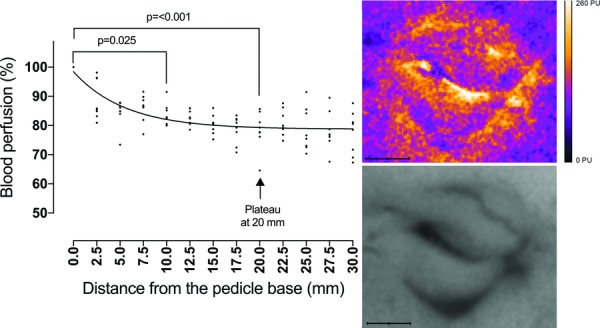
Laser speckle contrast imaging measurements showing a decrease in perfusion along the length of a full-thickness eyelid flap as the percent decrease in blood perfusion at increasing distance from the pedicel base. Nonlinear regression analysis showed that perfusion reached a plateau and stabilized at 20 mm from the base (95% CI, 16-23). Statistical analysis was performed using the Friedman matched-pair test with Dunn's posttest (n = 8). The images on the right are representative examples of the laser speckle pattern (top) and the corresponding grayscale image (bottom) of the upper and lower eyelids.

**Figure 3:**
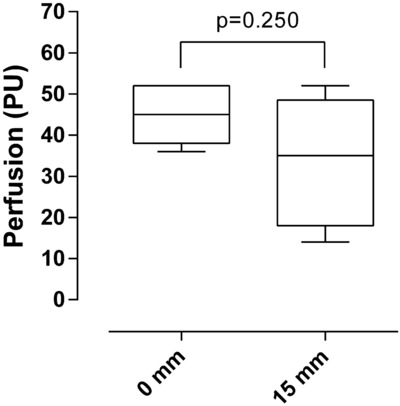
Laser Doppler velocimetry measurements of perfusion in a full-thickness eyelid flap on a pedicel, at 0 mm and 15 mm from the pedicel base (n = 5). Statistical analysis was performed using the Wilcoxon matched-pair test.

Perfusion in the pedicel base was 45 PU (range, 36-52) according to LDV and 110 PU (range, 105-145) according to LSCI. LSCI measurements showed that the blood flow decreased gradually from the pedicel base to the tip of the full-thickness eyelid flap; most of the decrease being seen over the first 10 mm (95 PU at 10 mm; range, 87-116 PU; *P* = .025, compared with that in the pedicel base). Perfusion reached a plateau and stabilized at 20 mm from the base (85 PU at 20 mm; range, 73-117; *P* < .001, compared with the pedicel base). No further decrease in perfusion was seen beyond this point (90 PU at 30 mm; range, 72-116; n = 8; Fig 2). LDV measurements confirmed these results, showing a decrease in perfusion of 15 mm from the pedicel base (Fig 3).

**Figure 4:**
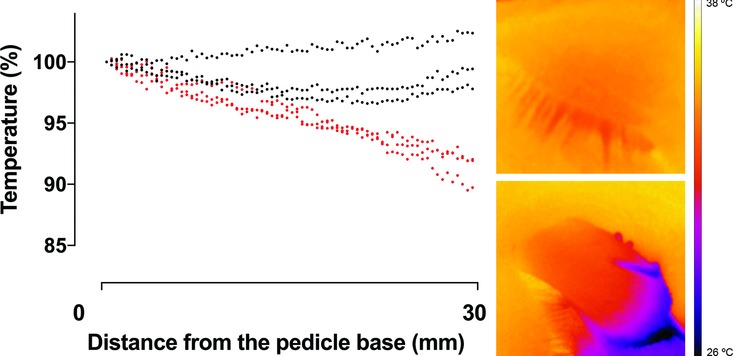
Thermographic measurements showing a decrease in temperature from the pedicel base to the tip of the full-thickness eyelid flap (red symbols), compared with that of intact eyelids (black symbols), calculated as the percentage of the temperature in the pedicel base (n = 3). The images on the right are representative examples of thermographic images of an intact eyelid (top) and a dissected eyelid (bottom).

Thermography showed a decrease in tissue temperature over the length of the full-thickness eyelid flaps. The tissue temperature was 34.34°C (range, 32.91-35.17) in the pedicel base, 33.16°C (range, 32.36-33.93) 10 mm from the pedicel base, 32.51°C (range, 31.16-33.26) 20 mm from the pedicel base, and 33.57°C (range, 29.53-32.37) 30 mm from the pedicel base (Fig 4). Thermography measurements were only performed in 3 pigs to illustrate the change in temperature, rather than to provide the basis for statistical assessment. The results of the thermography measurements are in accordance with, and support, the results obtained using LDV and LSCI.

## DISCUSSION

Dividing the eyelid from its blood supply is common during blepharotomy procedures, entropion repair with, for example, a Quickert procedure,^1^ or reconstructive procedures after tumor surgery, for example, Tenzel flap,^2^ or “switch flap” that is a full-thickness eyelid flap on a pedicel.^3^ The results of the present study show a decrease in perfusion over the length of the full-thickness eyelid flaps. However, perfusion is as high as 83% at 10 mm from the pedicel base, 79% at 20 mm, and 80% at 30 mm, indicating good perfusion in the entire flap. The reason for this well-maintained perfusion is presumably that it is well vascularized with blood supply through the attachment at the lateral canthus, where a branch of the lateral palpebral artery supplies the tarsal arcades.^7^^-^9

On the contrary, it is well known that in skin flaps that are not composite grafts, there is a steep drop in perfusion along the length of the flap. In a previous study on random advancement flaps on the flank of the pig, perfusion was found to be 40% at 20 mm from the flap base.^10^ Full-thickness eyelid flaps include all layers (including the anterior and posterior lamellae with skin, the orbicularis muscle, tarsal plate, and conjunctiva) and vascular structures (including the tarsal plexus)^7^ and may therefore be well perfused despite its length. It is most probable that the dissection of a skin flap on the eyelid will have less perfusion as a result of less vasculature entering it. The rich vascular supply of the periorbital region is probably why full-thickness eyelid flap are so permissive, and ischemia and necrosis seldom occur postsurgically.^11^ It allows the use of surgical reconstructions whose design would be inappropriate in other areas of the body.^7^^-^9^,^^11^^-^13

The eyelid was divided so that it remained attached only at the lateral canthus. Indeed, most of the blood flow to the eyelids comes from the medial canthus.^7^ It cannot be deduced from the present study whether this was responsible for the good perfusion in the eyelid or what the result would have been if the flap had been dissected in the opposite direction, that is, extending from the medial canthus.

Studies on microvascular blood perfusion are difficult, as they are hampered by artifacts. It is therefore of the utmost importance that studies are conducted using multiple methods to confirm the results. In the present study, perfusion was measured using 2 laser-based methods, LDV and LSCI, and tissue temperature was visualized using thermography. Laser-based methods such as LDV and LSCI quantify the change in motion in a specific tissue volume, which is interpreted as tissue blood perfusion. This means that artifacts will be introduced by movement resulting from, for example, breathing, and care must be taken to eliminate all sources of motion error. Thermography has been used to study plastic reconstructive procedures performance on other parts on the body.^14^^-^17 The change in skin temperature is believed to be proportional to changes in microcirculation, but it may also be due to other metabolic processes in the cells such as inflammatory responses and thermoregulatory enzymes. The change in temperature following a sudden obstruction of blood perfusion following surgical dissection will therefore be seen after a certain delay compared with the change in perfusion seen when using LDV or LSCI. This delay was obvious during the measurements in the present study. The change in temperature was not as reliable as the change in perfusion measured with the laser-based methods. However, the possible limitations of the techniques used in the present study are of less importance in the interpretation of the results since we were interested in changes in perfusion compared with a reference point in the same object and at the same point in time, rather than measuring the actual perfusion. Furthermore, comparison of the results obtained with the 3 methods supports the results and conclusion drawn from the study.

The current study was conducted in an eyelid flap model using the healthy tissue of a pig. Clinically, surgical reconstruction using flaps is commonly performed following the removal of a tumor from the eyelid or to rectify entropion. There is a risk of impaired wound healing in the tissues of the eyelids due to inflammation, infection, or edema or, in the case of the elderly, the skin may be very thin. Diabetes and cardiovascular disease may also affect the local vascular status of the eyelid, as will smoking. Other factors that may jeopardize postoperative healing include hematoma, tension or kinking of the tissue, extensive previous surgery, and irradiation. Infection in the early postoperative period can destroy poorly vascularized tissues. Since the present study was conducted on healthy tissues in an experimental setting, the results should be interpreted with caution, as other local factors may affect flap survival in the clinical situation.

In conclusion, perfusion in a full-thickness eyelid flap was measured using LDV, LSCI, and high-resolution thermography. The results show that there is only a slight drop in perfusion over the length of the full-thickness eyelid flaps. In total, 80% perfusion is maintained even if the flap was 3 cm long. The good perfusion in the eyelid flaps may be due to the rich vascularization by the anastomotic network of blood vessels in the tarsal plate, which may increase the likelihood of flap survival and surgical success.
